# Flexural Capacity and Deflection of Fiber-Reinforced Lightweight Aggregate Concrete Beams Reinforced with GFRP Bars

**DOI:** 10.3390/s19040873

**Published:** 2019-02-20

**Authors:** Xi Liu, Yijia Sun, Tao Wu

**Affiliations:** School of Civil Engineering, Chang’an University, Xi’an 710061, China; sunyijiachd@163.com (Y.S.); wutao@chd.edu.cn (T.W.)

**Keywords:** fiber-reinforced polymer (FRP), lightweight aggregate concrete (LWC), steel fiber, balanced reinforcement ratio, ultimate moment, deflection

## Abstract

Adding fibers is highly effective to enhance the deflection and ductility of fiber-reinforced polymer (FRP)-reinforced beams. In this study, the stress and strain conditions of FRP-reinforced lightweight aggregate concrete (LWC) beams with and without fibers at ultimate load level were specified. Based on the sectional analyses, alternative equations to predict the balanced reinforcement ratio and flexural capacity for beams failed by balanced failure and concrete crushing were established. A rational equation for estimating the short-term stiffness of FRP–LWC beams at service-load levels was suggested based on Zhu’s model. In addition, the contribution of the steel fibers on the short-term stiffness was quantified incorporating the effects of FRP reinforcement ratio. The proposed short-term stiffness model was validated with measured deflections from an experimental database for fiber-reinforced normal weight concrete (FNWC) beams reinforced with FRP bars. Furthermore, six glass fiber-reinforced polymer (GFRP)-reinforced LWC beams with and without steel fibers were tested under four-point bending. Based on the test results, the proposed models and procedures according to current design codes ACI 440.1R, ISIS-M03, GB 50608, and CSA S806 were linked together by comparing their predictions. The results showed that increasing the reinforcement ratio and adding steel fibers decreased the strain of the FRP bars. The flexural capacity of the LWC beams with and without steel fibers was generally underestimated by the design codes, while the proposed model provided accurate ultimate moment predictions. Moreover, the proposed short-term stiffness model yielded reasonable estimations of deflection for both steel fiber-reinforced lightweight aggregate concrete (SFLWC) and FNWC beams.

## 1. Introduction

Steel reinforcement corrodes rapidly when exposed. With the urgency of the sustainable infrastructure development and the innovation of structural system, fiber-reinforced polymer (FRP) bar has become increasingly applied in engineering construction as a substitute to conventional steel reinforcement, due to its lightweight, high tensile strength and non-corrosive properties [1,2,3,4,5]. On the other hand, the artificial lightweight aggregates constitute promising alternatives to natural aggregates due to their environmentally-friendly production process [6]. Moreover, the reduced mass of lightweight aggregate concrete (LWC) allows for a reduction in cross section and reinforcement amount in the concrete structural members, and thus exerts a favorable effect on both seismic resistance and economy [7]. In view of the characteristics of FRP and fiber-reinforced lightweight aggregate concrete (FLWC), their combined use could be beneficial not only in improving the strength-to-weight ratio, but also in decreasing the overall life cost of the structures [8].

FRP bars display linear elastic behavior in tension until failure and exhibit no yielding. Therefore, for FRP-reinforced concrete (FRP-RC) beams, the over-reinforced section design is recommended by most of the design codes [9,10], since the failure mode of concrete crushing is less catastrophic than FRP rupture. However, the strong brittleness of the LWC could bring adverse impacts on the ductility performance of the FRP-RC beams. Furthermore, the FRP-RC beams tend to experience large deflection due to the low elastic modulus of the FRP bars. The incorporation of fibers in concrete has been proved to be effective in enhancing the deflection and ductility performance altogether [11]. It should be mentioned that the use of steel fibers in FRP-RC beams was found to be valid because the corrosion of the steel fibers had a marginal effect on the performance of the members, even for high concentrations of chloride solutions [12].

Numerous research studies have been conducted on the prediction of ultimate moment and deflection of FRP-RC beams reinforced with steel fibers. Yang et al. [13] investigated the accuracy of ultimate capacity models based on test results of carbon fiber-reinforced polymer (CFRP)-reinforced beams with steel fibers and confirmed that the ultimate moment could be correctly estimated by ACI 544.4R and Campione when the ultimate concrete compressive strain was assumed as 0.004. Issa et al. [14] compared the test results of fibrous glass fiber-reinforced polymer (GFRP)-reinforced concrete beams to the available models and concluded that the ultimate moment capacity was strongly underestimated by ACI 440.1R, while the effective moment of inertia equation proposed by Faza and Ganga Rao [15] yielded the best deflection predictions through comparison. Yoo et al. [16] carried out flexural tests on ultra-high-performance fiber-reinforced concrete beams reinforced with GFRP bars, and modified the deflection model in ACI 440.1R considering the fiber reinforcement in the tensile zone after cracking.

Up to now, research concerning bearing capacity and deformation of FRP-reinforced beams fabricated using LWC, especially FLWC, was still limited. This paper aims to establish the balanced reinforcement ratio, ultimate moment and deflection equations of LWC beams with and without fibers.

## 2. Balanced Reinforcement Ratio and Ultimate Moment Model of FRP–FLWC Beams

### 2.1. Strain and Stress Conditions

**Figure 1:**
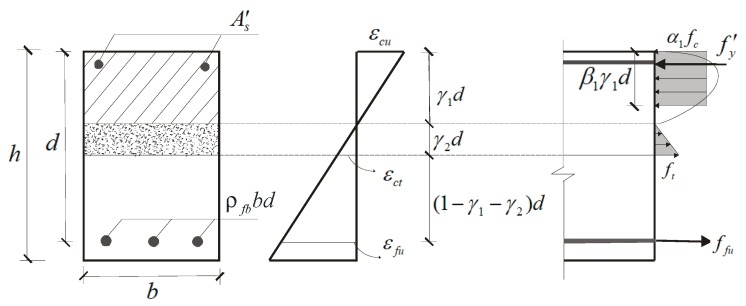
Strain and stress conditions for balanced failure mode.

The balanced failure refers to the failure mode in which the FRP tendons reach their ultimate tensile strain (*ε_fu_*) and the concrete reaches its ultimate compressive strain (*ε_cu_*) simultaneously. The corresponding reinforcement ratio is the balanced reinforcement ratio (*ρ_fb_*), which is the theoretical demarcation point to identify the failure mode of FRP-RC flexural members. At maximum load levels, FRP-reinforced beams generally experienced significant flexural cracking, although the fibers were added in the concrete [13,17]. Considering the fact that the fibers were pulled out or ruptured in cracked section, the tensile loads could not be transferred across the cracks by the bridging of fibers. Therefore, it is assumed that the concrete tensile stress in pre-cracking tensile zone (*γ*_2_*d*) distributes linearly, while concrete tensile strength in cracked section (*d*(1 − *γ*_1_ − *γ*_2_)) is ignored (Figure 1). In addition, the following assumptions are made for the calculation of the *ρ_fb_* and the ultimate moment (*M_u_*) of FRP–FLWC beams:

(1) Strain in the concrete and the FRP reinforcement is proportional to the distance from the neutral axis;
(2) The tensile behavior of the FRP reinforcement is linearly elastic;
(3) The stress-strain relationships of the FLWC under uniaxial tension and compression are assumed according to ACI 544.4R [18] and JGJ12 [19], respectively;
(4) The reinforcing steel rebar in the compression zone is yielded;
(5) A perfect bond exists between concrete and FRP reinforcement.

### 2.2. Prediction for Balanced Failure Mode

Based on the strain and stress conditions for balanced failure mode displayed in Figure 1, the *ρ_fb_* can be determined using strain compatibility approach:

(1) $$\begin{array}{l} \alpha_{1}\beta_{1}\gamma_{1}f_{c}bd+A_{s}^{\prime }f_{y}^{\prime }=f_{fu}\rho_{fb}bd+\frac{\gamma_{2}f_{t-F}bd}{2}\\ \frac{\varepsilon_{cu}}{\gamma_{1}d}=\frac{\varepsilon_{ct}}{\gamma_{2}d}=\frac{\varepsilon_{cu}+\varepsilon_{fu}}{d} \end{array}\}$$

where $A_{s}^{\prime }$ is the area of compressive reinforcement; *b* is the beam width; *d* is the beam effective depth; *f_c_* is the prism compressive strength of concrete; *f_fu_* is the tensile strength of the FRP bars; *f_t−F_* is the tensile strength of the fiber-reinforced concrete; $f_{y}^{\prime }$ is the yield strength of the steel rebars; *ε_ct_* is the peak tensile strain of the concrete; *ε_cu_* is the ultimate compressive strain of the concrete which assumed to be 0.0033 according to GB 50608 [20]; *γ*_1_ is the coefficient of neutral axis depth; *γ*_2_ is the coefficient of effective tensile concrete depth; *α*_1_, *β*_1_ are the stress-block factors for concrete, which can be determined by Equation (2), respectively.

(2) $$\begin{array}{l} \alpha_{1}=1-0.002\left(f_{cu}-40\right)\\ \beta_{1}=0.75-0.001\left(f_{cu}-40\right) \end{array}\}$$

where *f_cu_* is the cube compressive strength of concrete.

Combining Equations (1) and (2), the unknown value *γ*_1_ and *γ*_2_ can be obtained. Thereafter, the *ρ_fb_* and the *M_u_* can be expressed by Equations (3) and (4), respectively.

(3) $$\rho_{fb}=\frac{1}{f_{fu}}\left(\alpha_{1}\beta_{1}\gamma_{1}f_{c}+\frac{A_{s}^{\prime }f_{y}^{\prime }}{bd}-\frac{\gamma_{2}f_{t-F}}{2}\right)$$

(4) $$M_{u}=\alpha_{1}\beta_{1}\gamma_{1}f_{c}bd^{2}\left(1-\frac{\beta_{1}\gamma_{1}}{2}\right)+A_{s}^{\prime }f_{y}^{\prime }\left(d-a_{s}^{\prime }\right)-\frac{\gamma_{2}f_{t-F}bd^{2}}{2}\left(1-\gamma_{1}-\frac{2\gamma_{2}}{3}\right)$$

where $a_{s}^{\prime }$ is the concrete cover measured from the centroid of compressive reinforcement to the extreme compressive surface.

### 2.3. Prediction for Concrete Crushing Failure Mode

**Figure 2:**
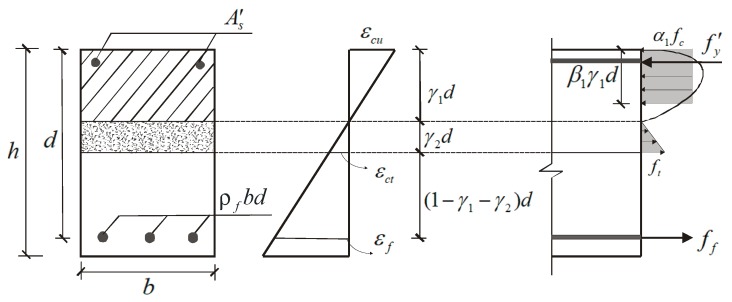
Strain and stress conditions for concrete crushing failure mode.

For beams that are damaged due to concrete crushing, based on the strain and stress conditions displayed in Figure 2, strain compatibility and force equilibrium are considered as follows:

(5) $$\begin{array}{l} \alpha_{1}\beta_{1}\gamma_{1}f_{c}bd+A_{s}^{\prime }f_{y}^{\prime }=\rho_{f}f_{f}bd+\frac{\gamma_{2}f_{t-F}bd}{2}\\ \frac{\varepsilon_{cu}}{\gamma_{1}d}=\frac{\varepsilon_{ct}}{\gamma_{2}d}=\frac{\varepsilon_{f}}{\left(1-\gamma_{1}\right)d} \end{array}\}$$

where *f_f_* is the tensile stress of the FRP bars; *ε_f_* is the tensile strain of the FRP bars; *ρ_f_* is the reinforcement ratio of the FRP bars.

As for the ultimate moment, Equation (4) can be also employed in the concrete crushing case since similar simplification of the stress distribution of concrete in the tension and compression zone is adopted.

## 3. Deflection Model of FRP-Reinforced Beams

### 3.1. Prediction for Beams without Fibers

**Figure 3:**
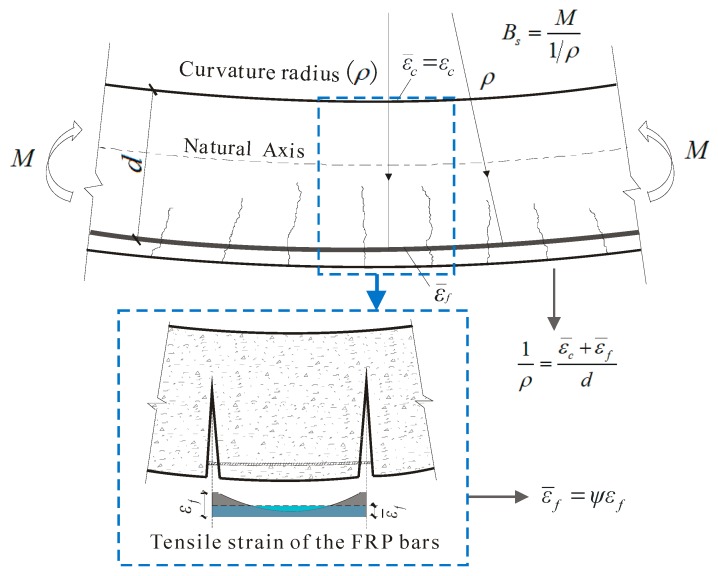
Calculation of curvature based on the fiber-reinforced polymer (FRP) tensile strain.

The curvature of FRP-RC members can be calculated based on the *ε_f_*, considering its nonuniform distribution between cracks, as shown in Figure 3.

(6) $$\frac{1}{\rho }=\frac{{\bar{\varepsilon }}_{f}+{\bar{\varepsilon }}_{c}}{d}=\frac{\psi \varepsilon_{f}+\varepsilon_{c}}{d}$$

where *ε_c_* is the compressive strain of the concrete; ${\bar{\varepsilon }}_{c}$ is the average compressive strain of the concrete; ${\bar{\varepsilon }}_{f}$ is the average tensile strain of the FRP bars; *ψ* is the nonuniformity coefficient of strain.

In GB 50608, the semi-empirical equation of the short-term stiffness (*B_s_*) was established by solving first the curvature (Equation (7)), based on the stiffness analytic method shown in Figure 3.

(7) $$B_{s}=\frac{E_{f}A_{f}d^{2}}{\frac{\psi }{\eta }+0.2+6\alpha_{fE}\rho_{f}}$$

where *A_f_* is the area of tension FRP bars; *α_fE_* is the ratio of modulus of elasticity of FRP bars to modulus of elasticity of concrete; *E_f_* is the modulus of elasticity of the FRP bars; *η* is the coefficient of the internal lever arm length, which is 0.87 and 0.85 for normal weight concrete and LWC beams, respectively. The *ψ* was given as

(8) $$\psi =1.1-0.65\frac{\eta A_{f}f_{t}d}{M\rho_{te}}$$

where *f_t_* is the tensile strength of concrete; *M* is the actual bending moment; *ρ*_te_ is the reinforcement ratio of the FRP bars on the basis of the effective cross-sectional area of concrete. It should be mentioned that the empirical parameters employed in Equations (7) and (8) were the same as those for steel reinforced beams.

Since the parameter *ψ* was included to reflect the variation in strain along the longitudinal reinforcement, accounting for the distinctive material properties of FRP reinforcements as compared with steel rebars, the *ψ* in Equation (8) might not be applicable to beams reinforced with FRP bars. Through regression analysis on experimental deflections of GFRP-reinforced flexural members at service-load levels, the empirical formula of the nonuniformity coefficient of strain in FRP bars (*ψ_f_*) was presented in the following Equation [21]:

(9) $$\psi_{f}=1.3-0.74\frac{\eta A_{f}f_{t}d}{M\rho_{te}}$$

By substituting Equation (9) into Equation (7), the short-term stiffness of beams reinforced with FRP bars (*B_s,f_*) can be expressed by

(10) $$B_{s,f}=\frac{E_{f}A_{f}d^{2}}{\frac{1.3}{\eta }-0.74\frac{A_{f}f_{t}d}{M\rho_{te}}+0.2+6\alpha_{fE}\rho_{f}}$$

### 3.2. Prediction for Beams Reinforced with Fibers

It has been proven that the concrete tensile strength increases with the volume fraction (*ρ_fi_*) and aspect ratio (*l_fi_*/*d_fi_*) of the fibers [22]. Based on this, the conversion between the tensile strengths of the concrete with and without fibers can be performed according to Equation (11).

(11) $$f_{t-F}=f_{t}\left(1+\alpha_{t}\lambda_{fi}\right)$$

where *α_t_* is the influence coefficient of fiber on the tensile strength of the concrete; *λ_fi_* is the feature parameter of fiber, which can be calculated by

(12) $$\lambda_{fi}=\frac{\rho_{fi}l_{fi}}{d_{fi}}$$

where *l_fi_* and *d_fi_* are the length and the diameter of fiber, respectively.

However, in the case of FRP-RC beams, it was observed that the improvement of their stiffness from the bridging effect of the fibers was reduced with the increasing reinforcement ratio [23]. With this in mind, the form of the amplification factor (1 + *α_t_λ_f_*) in Equation (11) is modified and the stiffness of FRP–FLWC beams (*B_s,f−F_*) can be expressed as

(13) $$B_{s,f-F}=\left(1+\alpha_{t}\lambda_{fi}\frac{\rho_{fb}}{\rho_{f}}\right)B_{s,f}$$

Based on the tensile tests on the plain and fibrous LWC, the *α_t_* can be determined by Equation (14), which is derived from Equation (11).

(14) $$\alpha_{t}=\frac{f_{t-F}-f_{t}}{\lambda_{fi}f_{t}}$$

### 3.3. Verification Based on Fiber-Reinforced Normal Weight Concrete (FNWC) Beams

**Figure 4:**
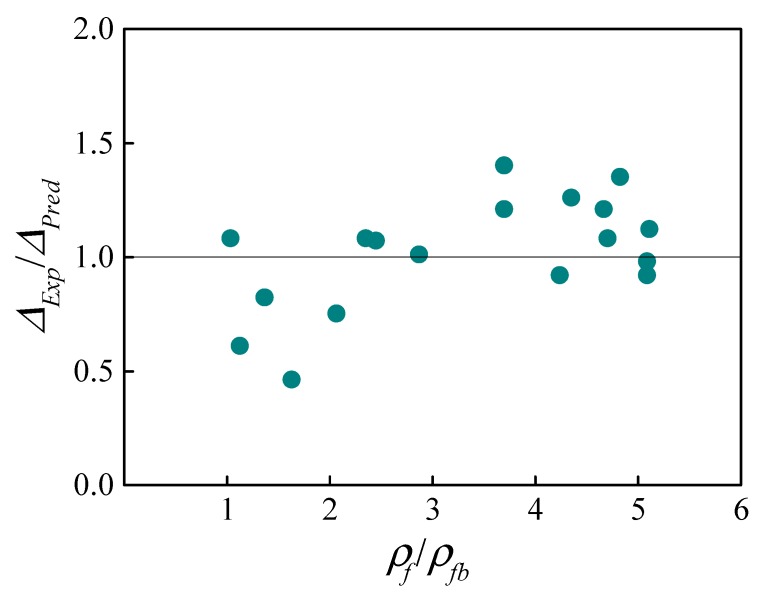
Ratios of experimental-to-predicted deflections for fiber-reinforced normal weight concrete (FNWC) beams.

In order to evaluate the accuracy of the short-term stiffness model proposed, the measured and predicted deflections under service load levels (*M_s_*) of FNWC beams reinforced with FRP bars tested in the literature are compared in Table 1 and Figure 4. It should be mentioned that the tensile strength of the concrete was ignored in the calculation of *ρ_fb_* due to its absence in most literature. Furthermore, since the ratio between the cracking moment of the beams was considered close to that between the tensile strength of the concrete used [23], the factor (*f_t−F_* − *f_t_*)/*f_t_* in Equation (14) was replaced by (*M_cr−F_* − *M_cr_*)/*M_cr_*, where *M_cr−F_* and *M_cr_* are the cracking moments of the FRP-reinforced beams with and without fibers, respectively.

The results in Figure 4 indicated that the estimations were generally in good agreement with the experimental results, although the definition of the service load given by the researchers was not unified. However, specimens with higher *ρ_f_/ρ_fb_* tended to yield higher experimental-to-predicted deflection ratios (*Δ_Exp_*/*Δ_Pred_*). It can be derived that the influence of *ρ_f_/ρ_fb_* on the *B_s,f−F_* presented in Equation (13) was overestimated.

## 4. Models Recommended by Design Codes

### 4.1. Balanced Reinforcement Ratio and Ultimate Moment

Strain compatibility approaches were used to calculate *ρ_fb_* and *M_u_* in current design codes. The *ρ_fb_* was determined from Equation (15) for ACI 440.1R and CSA S806, and Equation (16) for GB 50608, respectively. Moreover, stress-block factors for concrete (*α*_1_, *β*_1_) were computed by Equation (2), Equation (17) and Equation (18) for GB 50608, ACI 440.1R and CSA S806, respectively.

(15) $$\rho_{fb}=\alpha_{1}\beta_{1}\frac{f_{c}^{\prime }}{f_{fu}}\frac{E_{f}\varepsilon_{cu}}{E_{f}\varepsilon_{cu}+f_{fu}}$$

(16) $$\rho_{fb}=\frac{\alpha_{1}f_{c}}{f_{fu}}\frac{\beta_{1}\varepsilon_{cu}}{\varepsilon_{cu}+f_{fu}/E_{f}}$$

(17) $$\begin{array}{l} \alpha_{1}=0.85\\ \beta_{1}=0.85-\frac{0.05\left(f_{c}^{\prime }-28\right)}{7}\geq 0.65 \end{array}\}$$

(18) $$\begin{array}{l} \alpha_{1}=0.85-0.0015f_{c}^{\prime }\geq 0.67\\ \beta_{1}=0.97-0.0025f_{c}^{\prime }\geq 0.67 \end{array}\}$$

In ACI 440.1R, the *M_u_* is given by Equation (19) when *ρ_f_* ≥ *ρ_fb_*

(19) $$M_{u}=\rho_{f}f_{f}\left(1-0.59\frac{\rho_{f}f_{f}}{f_{c}^{\prime }}\right)bd^{2}$$

where the tensile strength of FRP bars (*f_f_*) could be calculated using the following equation:

(20) $$f_{f}=\left[\sqrt{\frac{{\left(E_{f}\varepsilon_{cu}\right)}^{2}}{4}+\frac{0.85\beta_{1}f_{c}^{\prime }}{\rho_{f}}E_{f}\varepsilon_{cu}}-0.5E_{f}\varepsilon_{cu}\right]\leq f_{fu}$$

In ISIS M03 [26], the *M_u_* for beams with *ρ_f_* ≥ *ρ_fb_* could be obtained according to Equation (21).

(21) $$M_{u}=\alpha_{1}\beta_{1}f_{c}^{\prime }bc\left(d-\frac{\beta_{1}c}{2}\right)$$

where *c* is the neutral axis depth from the top compression fiber, which was determined using Equation (22) as follows:

(22) $$c=\frac{0.5A_{f}E_{f}\varepsilon_{cu}}{\alpha_{1}\beta_{1}f_{c}^{\prime }b}\left[\sqrt{1+\frac{4\alpha_{1}\beta_{1}f_{c}^{\prime }}{\rho_{f}E_{f}\varepsilon_{cu}}}-1\right]$$

Based on semiempirical considerations, GB 50608 recommended *M_u_* equation as:

(23) $$M_{u}\leq f_{f}A_{f}\left(d-\frac{f_{f}A_{f}}{2f_{c}b}\right)$$

The experienced formula of *f_f_* was expressed as a piecewise function:

(24) $$f_{f}=\{\begin{array}{cc} f_{fu}\left[1-0.211{\left(\frac{\rho_{f}}{\rho_{fb}}-1\right)}^{0.2}\right] & \mathrm{for}\;\rho_{fb}<\rho_{f}<1.5\rho_{fb}\\ f_{fu}{\left(\frac{\rho_{f}}{\rho_{fb}}\right)}^{-0.50} & \mathrm{for}\;1.5\rho_{fb}\leq \rho_{f} \end{array}$$

### 4.2. Deflection

In design codes GB 50608, ISIS-M03 and ACI 440.1R, the procedures to calculate the deflections of simply supported FRP-RC beams under four-point bending entailed the calculation of the average stiffness throughout the beam length.

(25) $$\Delta =\frac{Pa}{48A}\left(3L^{2}-4a^{2}\right)$$

where *a* is the beam shear span; *P* is the applied concentrated load; *Δ* is the midspan deflection; *A* represents the beam average stiffness, which is *B*_s_ in GB 50608, and is the product of the elastic modulus of the concrete (*E*_c_) and the effective moment of inertia (*I_e_*) in ISIS-M03 and ACI 440.1R.

ACI 440.1R employed the *I_e_* in predicting the deflection of FRP-RC beams. The formula for *I_e_* was modified Bischoff’s expression [27,28]:

(26) $$I_{e}=\frac{I_{cr}}{1-\gamma {\left(\frac{M_{cr}}{M_{a}}\right)}^{2}\left[1-\frac{I_{cr}}{I_{g}}\right]}$$

where *I_cr_* is the cracked moment of inertia; *I_g_* is the gross moment of inertia; *M_a_* is the actual bending moment; The reduction factor *γ* was included to account for the variation in stiffness along the length of the member

(27) $$\gamma =1.72-0.72\left(\frac{M_{cr}}{M_{a}}\right)$$

The alternative expression for *I_e_* suggested by ISIS-M03 was confirmed to be conservative over the entire range of the test specimens by Mota et al. [29]:

(28) $$I_{e}=\frac{I_{t}I_{cr}}{I_{cr}+\left(1-0.5{\left(\frac{M_{cr}}{M_{a}}\right)}^{2}\right)\left(I_{t}-I_{cr}\right)}$$

where *I_t_* is the second moment of area of the uncracked section transformed to concrete.

CSA S806 predicted the deflection of FRP-RC members using moment–curvature method:

(29) $$\Delta =\frac{PL^{3}}{24E_{c}I_{cr}}\left[3\left(\frac{a}{L}\right)-4{\left(\frac{a}{L}\right)}^{2}-8\left(1-\frac{I_{cr}}{I_{g}}\right){\left(\frac{L_{g}}{L}\right)}^{3}\right]$$

where *L_g_* is the uncracked beam length.

## 5. Experimental Study of GFRP-Reinforced LWC Beams

### 5.1. Test Specimens and Material Properties

**Figure 5:**
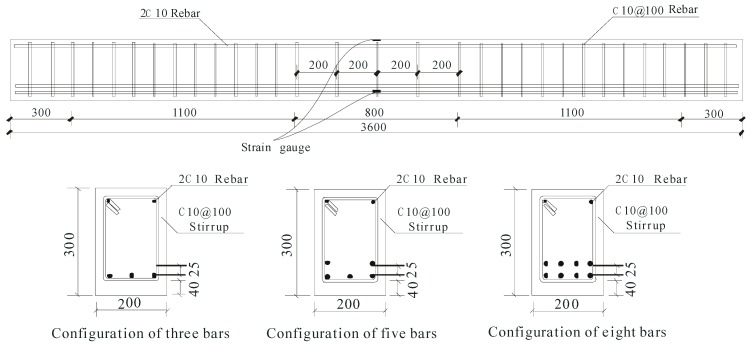
Specimen details (dimensions in millimeters).

**Figure 6:**
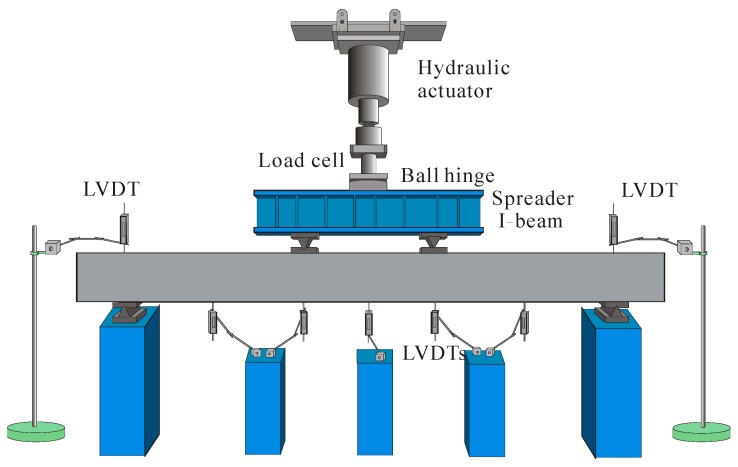
Test setup.

To study the influence of steel fibers and FRP reinforcement ratio, six GFRP-reinforced LWC beams were constructed and tested under four-point bending. Beam geometry and the loading and support arrangement are illustrated in Figure 5 and Figure 6, respectively. Strain gauges were installed on the FRP reinforcement at midspan and on the top surface of the concrete in the constant moment region to monitor their strain during the loading process.

LWC with steel fiber contents of 0% and 0.6%, respectively, were used to fabricate the specimens. The properties of steel fibers are listed in Table 2. The cube compressive strength *f_cu_* were obtained from testing three 100 mm cubes for each beam on the day of testing. The tensile strengths of the LWC and steel fiber-reinforced lightweight aggregate concrete (SFLWC) were 5.12 MPa and 8.16 MPa, respectively.

Sand coated GFRP bars with a diameter of 13.77 mm were used as longitudinal reinforcements. The diameters and mechanical properties of the GFRP bars were determined according to ACI 440.3R [30]. Steel rebars with diameter, tensile strength and elastic modulus of 10.62 mm, 445 MPa and 204 GPa, respectively, were used as transverse and top reinforcements. Table 3 presents the mechanical properties of the GFRP bars and the details of the tested specimens.

The beams were labeled as following: LC or SL for lightweight aggregate concrete or steel fiber-reinforced lightweight aggregate concrete; then G for GFRP bars; finally, the reinforcement ratio (percent). For instance, LCG–0.92 indicates a lightweight aggregate concrete beam with GFRP reinforcement ratio of 0.92%.

### 5.2. Test Results

**Figure 7:**
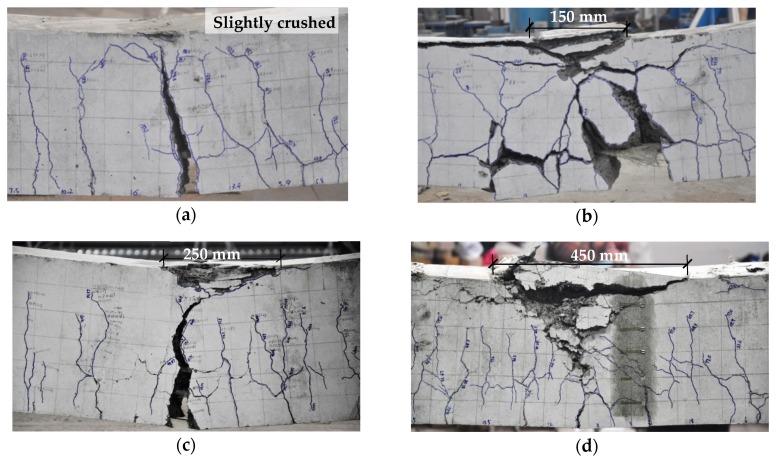
Typical failure shapes: (**a**) SLG–0.92; (**b**) LCG–0.92; (**c**) SLG–1.64; (**d**) SLG–2.66 (dimensions in millimeters).

**Figure 8:**
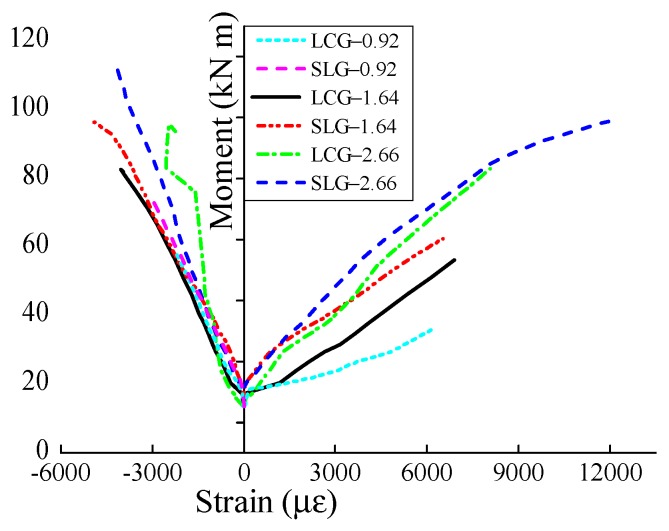
Moment–FRP and concrete strain relationships.

The test results are shown in Table 3, and the typical failure shapes of the specimens are depicted in Figure 7. Crushing of lightweight aggregates (LWAs) could be observed at the fractured surface in the compression zone (Figure 7b–d), owing to their low compressive strength. This was a distinctive fracture pattern of LWC beams as compared with the ordinary concrete ones. Figure 8 shows the strains in the tensile FRP bars and the compressive concrete against the applied moment of the specimens. The reinforcement strain of beam SLC–0.92 is not presented since the strain gauges were broken down. Both the moment–FRP and concrete strain responses could be divided into two segments. The first stage was a steep linear curve corresponding to the behavior before cracking, wherein the FRP bars and concrete exhibited little strain, while the second was a basically linear segment represented the post-cracking behavior. In addition, increasing the reinforcement ratio and adding steel fibers could restrain the deformation of the FRP bars, indicating their benefit achieved in flexural stiffness of the beams. Figure 8 also illustrates that the concrete strain at the maximum load level generally exceeded the assumed concrete compressive strain recommended by GB 50608.

## 6. Comparison between the Experimental and Predicted Results

### 6.1. Balanced Reinforcement Ratio and Ultimate Moment of LWC Beams

The relative reinforcement ratio *ρ_f_/ρ_fb_* of the tested specimens according to GB 50608, CSA S806, ACI 440.1R and Equation (3) are presented in Table 4. It is to be noted that the tensile strength of the LWC without steel fibers was ignored. The results indicated that specimens with *ρ_f_/ρ_fb_* close to 1 all possibly damaged by balanced failure, which could be attributed to the discreteness of material strength. In addition, the greater *ρ_f_/ρ_fb_* lent a higher degree of concrete crushing when FRP bars ruptured (Figure 7a–c). As presented in Table 4, CSA S806 provided more conservative *ρ_f_/ρ_fb_* than ACI 440.1R although the same *ρ_fb_* equation was used. This could be ascribed to the higher concrete compressive strain assumed. Additionally, the proposed model provided the lowest *ρ_f_/ρ_fb_* among all the models since the contribution of the compressive reinforcements was included.

**Figure 9:**
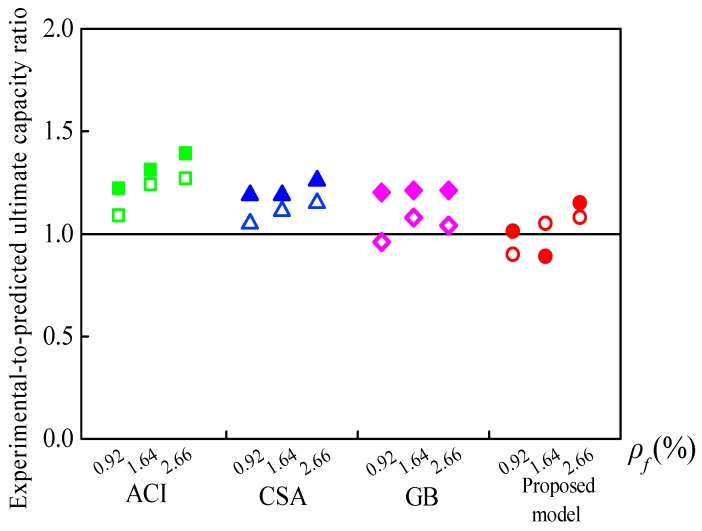
Ratios of experimental-to-predicted ultimate capacity.

Based on the actual failure mode, the experimental-to-predicted ultimate capacity ratios (*M_u,Exp_*/*M_u,Pred_*) of the specimens according to design codes and the proposed model are summarized in Table 4. Unfilled and filled points are used to represent the *M_u,Exp_*/*M_u,Pred_* of the LWC and SFLWC specimens in Figure 9, respectively. As shown in Figure 9, the estimated *M_u_* according to the design codes were generally lower than the experimental results since the contribution of the steel fibers and the reinforcement in the compression zone was ignored.

For LWC beams, the average *M_u,Exp_*/*M_u,Pred_* based on GB 50608, CSA S806, and ACI 440.1R were 1.06 ± 0.07, 1.10 ± 0.05, 1.20 ± 0.1, respectively. Additionally, the predictions obtained from the proposed model were in good agreement with the experimental results with an average *M_u,Exp_*/*M_u,Pred_* of 1.01 ± 0.10.

In the case of the SFLWC specimens, GB 50608, CSA S806, and ACI 440.1R provided conservative ultimate capacities with average *M_u,Exp_*/*M_u,Pred_* of 1.19 ± 0.01, 1.21 ± 0.04, 1.31 ± 0.09, respectively. On the other hand, the proposed model yielded most accurate predictions with an average *M_u,Exp_*/*M_u,Pred_* of 1.02 ± 0.13.

As a perfect bond between concrete and FRP reinforcement was assumed, the deformation caused by relative slip between the LWC and the FRP reinforcement was ignored. Based on the plane section assumption, the actual depth of the compression zone was lower than the calculated value. Therefore, this assumption would result in overestimation of the ultimate capacity.

### 6.2. Deflection of LWC Beams

**Figure 10:**
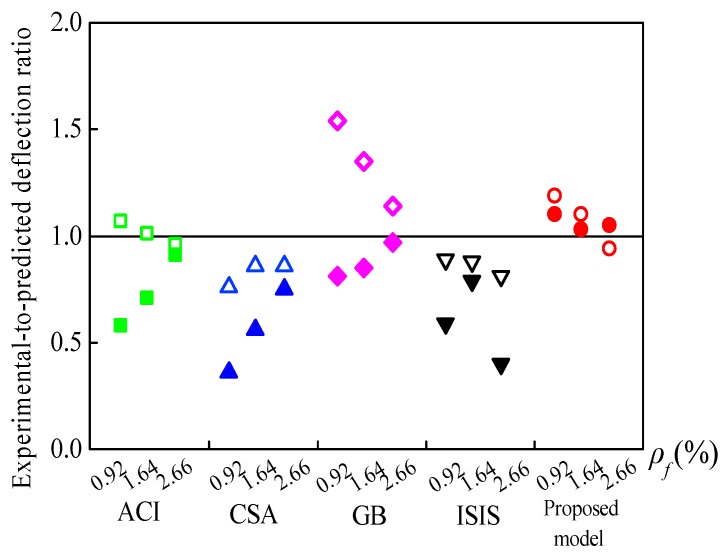
Ratios of experimental-to-predicted deflections for lightweight aggregate concrete (LWC) beams.

Bischoff et al. [31] recommended a load corresponding to 30% of the *M_u_* as service load (*M_s_*) for FRP-RC beams. Table 5 and Figure 10 compare the experimental deflections with the theoretical results estimated by design codes ACI 440.1R, CSA S806, ISIS-M03 and GB 50608 and the proposed model. The *Δ_Exp_*/*Δ_Pred_* of the LWC and SFLWC specimens with varied *ρ_f_* are represented by unfilled and filled points in Figure 10, respectively. For LWC beams, CSA S806 and ISIS-M03 overestimated the deflections with average *Δ_Exp_*/*Δ_Pred_* of 0.83 ± 0.06 and 0.86 ± 0.04, respectively. On the other hand, GB 50608 gave unconservative predictions with an average *Δ_Exp_*/*Δ_Pred_* of 1.34 ± 0.20. Moreover, ACI 440.1R and the proposed model showed accurate predictions with average *Δ_Exp_*/*Δ_Pred_* of 1.01 ± 0.06 and 1.07 ± 0.13, respectively.

In the case of the SFLWC specimens, the measured results were generally lower compared to the predicted deflections based on the design codes, owing to the benefits offered by the steel fibers. At *M_s_*, CSA S806 and ISIS-M03 provided significantly conservative estimations with average *Δ_Exp_*/*Δ_Pred_* of 0.56 ± 0.20 and 0.59 ± 0.20, respectively. ACI 440.1R and GB 50608 slightly overestimated the deflections with average *Δ_Exp_*/*Δ_Pred_* of 0.73 ± 0.17 and 0.88 ± 0.08, respectively. Furthermore, it was confirmed that the deflections could be reasonably estimated by the proposed model with an average *Δ_Exp_*/*Δ_Pred_* of 1.06 ± 0.04. Similarly, the results in Figure 10 could be taken as an evidence for the overestimation of the influence of *ρ_f_/ρ_fb_* in Equation (12). Considering the overall predictions of LWC and SFLWC beams, the proposed model provided the lowest COV among the equations used, namely, 7%.

**Figure 11:**
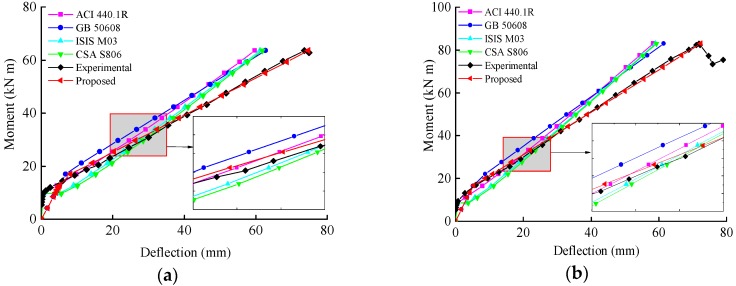

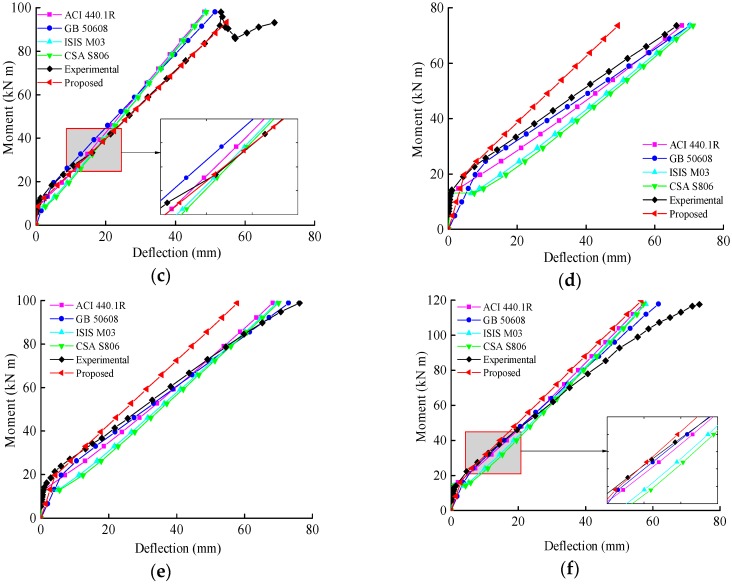
Comparison between the experimental and theoretical moment–deflection responses (**a**) LCG–0.92; (**b**) LCG–1.64; (**c**) LCG–2.66; (**d**) SLG–0.92; (**e**) SLG–1.64; (**f**) SLG–2.66.

Figure 11 compares the experimental and theoretical moment–deflection responses of the tested specimens. As the figure shows, for the LWC beams, the proposed equation yielded similar load versus midspan deflection behaviors with the test data during the entire loading period. However, for beams reinforced with steel fibers, the proposed model yielded accurate estimations only at service-load levels. This could be attributed to the fact that the improvement of the stiffness from the steel fibers gradually diminished as the fibers pulled out in succession at high-load levels.

## 7. Conclusions

This paper establishes the balanced reinforcement ratio, ultimate moment and deflection equations of LWC beams with and without fibers. The accuracy of the proposed models was investigated based on experimental results. The following conclusions can be drawn:

(1) Increasing the reinforcement ratio and adding steel fibers were shown to be effective in decreasing the FRP strain of FRP-reinforced LWC beams.
(2) Design codes ACI 440.1R, ISIS-M03, GB 50608, and CSA S806 generally underestimated the flexural capacity of the GFRP-LWC beams with and without steel fibers. The predictions obtained from the proposed ultimate moment equation were in good agreement with the experimental results.
(3) For GFRP-SFLWC beams, the design codes showed conservative deflection values at *M_s_*, while the proposed short-term stiffness model provided reasonable predictions with low dispersion degree.
(4) In both case of SFLWC and FNWC beams, specimens with higher *ρ_f_*/*ρ_fb_* tended to yield higher experimental-to-predicted deflection ratios based on the proposed model. Further research will be conducted involving rational consideration of the FRP reinforcement amount in the deflection model of fiber-reinforced beams.

## Figures and Tables

**Table 1:** Comparisons between the theoretical and experimental deflections of FNWC beams at *M_s_*.

Reference	Spec.	Service Load	Type of Bar	Type of Fiber ^1^	*L* (mm)	*h* (mm)	*b* (mm)	$f_{c}^{\prime }$ (MPa)	*f_fu_* (MPa)	*E_f_* (GPa)	*ρ_f_* (%)	*ρ_f_*/*ρ_fb_*	*Δ_Exp_* (mm)	*Δ_Exp_*/*Δ_Pred_*
Wang and Belarbi [11]	F8G	0.3*M_u_*	GFRP	PP	1829	229	178	30	690	41	2.13	4.71	6.22	1.08
	F8G		GFRP	PP	1829	229	178	30	552	41	3.17	4.83	4.29	1.35
	F4C		CFRP	PP	1829	229	178	30	2069	124	0.64	4.24	4.64	0.92
Yang et al. [13]	CC-SN	0.3*M_u_*	CFRP	SYF	1900	250	230	89.3	2130	146.2	0.60	2.45	5.99	1.07
	CC-ST		CFRP	SF	1900	250	230	104.4	2130	146.2	0.60	2.87	4.28	1.01
	GG-SN		GFRP	SYF	1900	250	230	89.3	941	48.1	1.88	4.35	7.36	1.26
	GG-ST		GFRP	SF	1900	250	230	104.4	941	48.1	1.88	5.11	7.36	1.12
Issa et al. [14]	NP	0.4*M_u_*	GFRP	PP	1500	150	150	31.58	347.5	33	1.87	1.37	9.59	0.82
	HP		GFRP	PP	1500	150	150	51.42	347.5	33	1.87	1.04	8.65	1.08
	NG		GFRP	GF	1500	150	150	24.88	347.5	33	1.87	1.63	5.72	0.46
	HG		GFRP	GF	1500	150	150	43.62	347.5	33	1.87	1.13	7.12	0.61
	NS		GFRP	SF	1500	150	150	18.38	347.5	33	1.87	2.07	11.5	0.75
Rashid et al. [24]	DF3T1F	*M_u_*/1.7	GFRP	PVA	2400	300	150	79.61	1760	53	0.59	3.7	33.8	1.40
	DF3T2F		GFRP	PVA	2400	300	150	79.61	1760	53	0.59	3.7	19.7	1.21
Saikia et al. [25]	FG1SFPC	0.4*M_u_*	GFRP	PP&P	1340	250	180	35.49	972	49	0.78	2.35	3.62	1.08
	FG1GFPC		GFRP	PP&P	1340	250	180	31.03	972	49	0.78	4.67	3.15	1.21
	FG2SFC	0.3*M_u_*	GFRP	PP	1340	250	180	30.23	972	49	1.51	5.09	1.93	0.98
	FG2GFC		GFRP	PP	1340	250	180	30.23	972	49	1.51	5.09	1.73	0.92
Average														1.02
Standard deviation														0.246
Coefficient variation (COV) (%)														24

^1^ PP is the polypropylene fiber; GF is the glass fiber; SF is the steel fiber; SYF is the synthetic fiber; PVA is the polyvinyl alcohol fiber; PP&P is the polypropylene fiber and polymer; PVA is the polyvinyl alcohol fiber. $f_{c}^{\prime }$ is the cylinder compressive strength of concrete; *h* is the beam height; *L* is the beam clear span; *Δ_Exp_* is the experimental deflection; *Δ_Pred_* is the predicted deflection.

**Table 2:** Properties of steel fiber.

Type	*l_fi_* (mm)	*d_fi_* (μm)	Density (kg·m^−3^)	Tensile Strength (MPa)	Shape	Surface
Steel fiber	13	200	7800	>3000	Straight	Smooth

**Table 3:** Summary of specimen details and test results.

Spec.	*f_f__u_* (MPa)	*E_f_* (GPa)	*f_cu_* (MPa)	*E_c_* (GPa)	*ρ_f_* (%)	*E_f_A_f_* (kN)	*M_u_* (kN·m)	Failure Mode ^1^	*Δ* (mm)	
									0.3*M_u_*	*M_u_*
LCG–0.92	602	44	54.92	31.2	0.92	19816	63.51	B.F	13.3	73.8
SLG–0.92	602	44	81.62	32.9	0.92	19816	73.54	B.F	7.1	64.9
LCG–1.64	602	44	64.68	31.2	1.64	33026	82.92	C.C	15.0	71.8
SLG–1.64	602	44	82.32	32.9	1.64	33026	98.75	B.F	11.3	76.2
LCG–2.66	602	44	62.52	31.2	2.66	52842	97.96	C.C	12.5	52.8
SLG–2.66	602	44	79.41	32.9	2.66	52842	119.85	C.C	13.1	76.8

^1^ B.F. and C.C. denote balanced and concrete crushing failure modes, respectively.

**Table 4:** Relative reinforcement ratios and experimental-to-predicted ultimate capacity ratios.

Spec.	*ρ_f_/ρ_fb_*				*M_u,Exp_*/*M_u,Pred_*			
	ACI 440.1R	CSA S806	GB 50608	Proposed Model	ACI 440.1R	CSA S806	GB 50608	Proposed Model
LCG–0.92	1.09	0.89	0.98	0.76	1.09	1.05	0.98	0.90
SLG–0.92	0.83	0.66	0.71	0.59	1.22	1.19	1.18	1.01
LCG–1.64	1.78	1.39	1.52	1.20	1.24	1.11	1.11	1.05
SLG–1.64	1.46	1.17	1.26	1.04	1.31	1.19	1.20	0.89
LCG–2.66	2.94	2.33	2.55	1.99	1.27	1.15	1.09	1.08
SLG–2.66	2.46	1.96	2.11	1.72	1.39	1.26	1.20	1.15
Average					1.25	1.16	1.13	1.02
Standard deviation					0.10	0.07	0.09	0.10
Coefficient of variation (COV) (%)					8	6	8	10

**Table 5:** Experimental-and-predicted deflections ratios for LWC beams.

Spec.	*Δ_Exp_*/*Δ_Pred_*				
	ACI 440.1R	CSA S806	GB 50608	ISIS-M03	Proposed Model
LCG–0.92	1.07	0.76	1.54	0.89	1.19
SLG–0.92	0.58	0.36	0.81	0.59	1.10
LCG–1.64	1.01	0.86	1.35	0.88	1.10
SLG–1.64	0.71	0.56	0.85	0.79	1.03
LCG–2.66	0.96	0.86	1.14	0.81	0.94
SLG–2.66	0.91	0.75	0.97	0.40	1.05
Average	0.87	0.69	1.11	0.79	1.07
Standard deviation	0.19	0.20	0.29	0.19	0.08
COV (%)	22	28	26	24	7

## References

[1] Dong Z., Wu G., Xu Y. (2016). Experimental study on the bond durability between steel-FRP composite bars (SFCBs) and sea sand concrete in ocean environment. Constr. Build. Mater..

[2] Zheng Y., Yu T., Yang J., Li Y., Sun C. (2017). Investigation of the behaviour of reinforcement-free concrete deck slabs restrained by FRP rods. Eng. Struct..

[3] Zheng Y., Li C., Yang J., Yu T., Sun C. (2015). Influence of arching action on shear behaviour of laterally restrained concrete slabs reinforced with GFRP bars. Compos. Struct..

[4] Xia L., Zheng Y. (2018). Deep embedment (DE) FRP shear strengthening of concrete bridge slabs under loads close to supports. Appl. Sci..

[5] Xu K., Ren C., Deng Q., Jin Q., Chen X. (2018). Real-time monitoring of bond slip between GFRP bar and concrete structure using piezoceramic transducer-enabled active sensing. Sensors.

[6] Kayali O. (2008). Fly ash lightweight aggregates in high performance concrete. Constr. Build. Mater..

[7] Bogas J.A., Brito J.D., Cabaço J. (2014). Long-term behaviour of concrete produced with recycled lightweight expanded clay aggregate concrete. Constr. Build. Mater..

[8] Li P., Sui L., Xing F., Huang X., Zhou Y., Yun Y. (2018). Effects of aggregate types on the stress-strain behavior of fiber reinforced polymer (FRP)-confined lightweight concrete. Sensors.

[9] ACI 440.1R (2015). Guide for the Design and Construction of Structural Concrete Reinforced with Fiber-Reinforced Polymer Bars.

[10] CSA S806 (2012). Design and Construction of Building Structures with Fibre-Reinforced Polymers.

[11] Wang H.Z., Belarbi A. (2013). Flexural durability of FRP bars embedded in fiber-reinforced-concrete. Constr. Build. Mater..

[12] Lambrechts A., Nemegeer D., Vanbrabant J., Stang H. (2003). Durability of steel fibre reinforced concrete. Spec. Publ..

[13] Yang J.M., Min K.H., Shin H.O., Yoon Y.S. (2012). Effect of steel and synthetic fibers on flexural behavior of high-strength concrete beams reinforced with FRP bars. Compos. Part. B Eng..

[14] Issa M.S., Metwally I.M., Elzeiny S.M. (2011). Influence of fibers on flexural behavior and ductility of concrete beams reinforced with GFRP rebars. Eng. Struct..

[15] Faza S.S., Ganga Rao H.V.S. (1990). Bending and bond behavior of concrete beams reinforced with plastic rebars. Transport. Res. Rec..

[16] Yoo D.Y., Banthia N., Yoon Y.S. (2016). Predicting service deflection of ultra-high-performance fiber reinforced concrete beams reinforced with GFRP bar. Compos. Part B.

[17] Zhu H.T., Cheng S.Z., Gao D.Y., Neaz S.M., Li C. (2018). Flexural behavior of partially fiber-reinforced high-strength concrete beams reinforced with FRP bars. Constr. Build. Mater..

[18] ACI 544.4R (2018). Guide to Design with Fiber-Reinforced Concrete.

[19] JGJ12-2006 (2006). Technical Specification for Lightweight Aggregate Concrete Structures.

[20] GB 50608 (2010). Technical Code for Infrastructure Application of FRP Composites.

[21] Zhu H., Dong Z.Q., Wu G., Wu Z.S. (2015). Experimental study and theoretical calculation on the flexural stiffness of concrete beams reinforced with FRP bars. Chin. Civ. Eng. J..

[22] Oh B.H. (1992). Flexural Analysis of Reinforced Concrete Beams Containing Steel Fibers. J. Struct. Eng..

[23] El-Nemr A., Ahmed E.A., Benmokrane B. (2013). Flexural behavior and serviceability of normal- and high-strength concrete beams reinforced with glass fiber-reinforced polymer bars. ACI Struct. J..

[24] Rashid M.A., Mansur M.A., Paramasivam P. (2005). Behavior of aramid fiber-reinforced polymer reinforced high strength concrete beams under bending. J. Compos. Constr..

[25] Saikia B., Kumar P., Thomas J., Rao K.S.N., Ramaswamy A. (2007). Strength and serviceability performance of beams reinforced with GFRP bars in flexure. Constr. Build. Mater..

[26] ISIS-M03 (2007). Reinforcing Concrete Structures with Fibre Reinforced Polymers.

[27] Bischoff P.H. (2005). Reevaluation of deflection prediction for concrete beams reinforced with steel and fiber reinforced polymer bars. J. Struct. Eng..

[28] Bischoff P.H., Gross S.P. (2011). Equivalent moment of inertia based on integration of curvature. J. Compos. Constr..

[29] Mota C., Alminar S., Svecova D. (2006). Critical review of deflection formulas for FRP-RC members. J. Compos. Constr..

[30] ACI 440.3R (2004). Guide Test Methods for Fiber-Reinforced Polymers (FRPs) for Reinforcing or Strengthening Concrete Structures.

[31] Bischoff P.H., Gross S., Ospina C.E. (2009). The story behind proposed changes to ACI 440 deflection requirements for FRP-reinforced concrete. Spec. Publ..

